# Effect of Social Support on Suicide Risk Among Posttraumatic Stress Disorder, Depressive, and Comorbid Symptom Groups in Elderly Living Alone in Korea

**DOI:** 10.1192/j.eurpsy.2026.10468

**Published:** 2026-07-31

**Authors:** M.-D. Kim, W.-M. Bahk, B.-H. Yoon, Y.-E. Jung

**Affiliations:** 1 Psychiatry, Jeju National University Hospital, Jeju; 2 Psychiatry, Woo and Bahk psychiatric clinic, Seoul; 3 Psychiatry, Naju National Hospital, Naju, Korea, Republic Of

## Abstract

**Introduction:**

With the rapid shift to ageing society, the number of elderly individuals living alone has been steadily increasing. This population is particularly vulnerable to psychiatric disorders including suicide. Jeju Island is a region where the entire population was collectively exposed to trauma, specifically the 4·3 Incident, a large-scale civilian massacre. The incident inflicted historical trauma on the entire community, with its psychological sequelae affecting not only survivors but also subsequent generations of bereaved families. Elderly individuals living alone, who lack both internal and external resources, may be especially susceptible to such psychiatric conditions including suicide.

**Objectives:**

This study aimed to classify elderly individuals living alone in Jeju into four groups based on their psychiatric status: a healthy group, a PTSD symptom group, a depressive symptom group, and a comorbid PTSD and depressive group. By comparing the effects of social support on suicide risk across these groups, this study proposes targeted suicide prevention strategies for elderly individuals living alone in Jeju.

**Methods:**

A complete enumeration survey of elderly individuals living alone in Jeju to examine the prevalence of suicide risk across four psychiatric groups-a healthy group, a PTSD symptom group, a depressive symptom group, and a comorbid group- and to compare the impact of social support on suicide risk among these groups. Of 5,138 eligible individuals, 4,680 participated in face-to-face interviews using structured questionnaires. PTSD symptoms were assessed with the PC-PTSD-5, depressive symptoms with the SGDS-K, suicide risk with the MINI-Plus, and social support with the MOS-SSS. Multivariate logistic regression was performed to find the effect of social support on suicide risk.

**Results:**

Among 4,680 participants, 7.8% reported PTSD symptoms, 40.4% reported depressive symptoms, and 5.8% had comorbid symptoms. Prevalence of suicide risk was 10.2% overall, with the highest rate observed in the comorbid group (29.5%), followed by the depressive (17.1%) and PTSD symptom groups (12.8%). Social support was significantly associated with lower suicide risk in the healthy (OR = 0.976, p < 0.001), depressive (OR = 0.980, p < 0.001), and comorbid groups (OR = 0.979, p < 0.001), but not in the PTSD symptom group (OR = 0.957, p = 0.179).

**Conclusions:**

Developing more tailored social support systems is essential to prevent suicide among individuals living alone who are vulnerable to psychiatric illness.

**Disclosure of Interest:**

None Declared

